# Supplementary material for: Proteomics Evidence of a Systemic Response to Desiccation in the Resurrection Plant Haberlea rhodopensis
Source: Int J Mol Sci. 2022 Jul 31;23(15):8520. doi: 10.3390/ijms23158520 (PMC9369045; doi:10.3390/ijms23158520)
Supplement: Supplementary file 1 [file ijms-23-08520-s001.zip › Supplementary figures.pdf]

SUPPLEMENTARY FIGURES

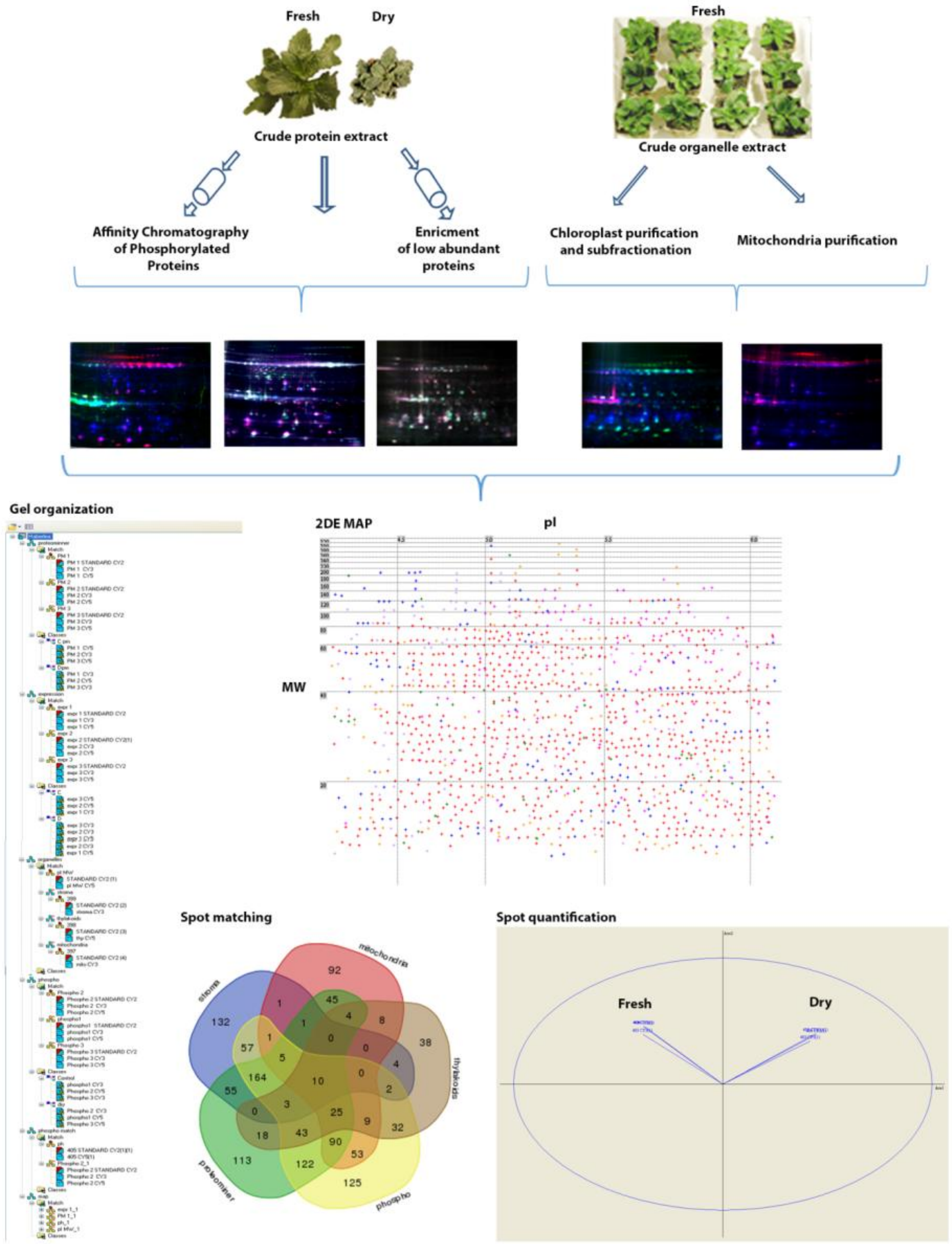

Figure S1. 2D DIGE Workflow. Organelle and protein extraction and fractionation approaches and the corresponding 2DE gels are shown. Gel files were next organized in project in ImageMaster 2D Platinum software, and spots were matched, quantified, and organized in 2DE proteome map according to internal standard.

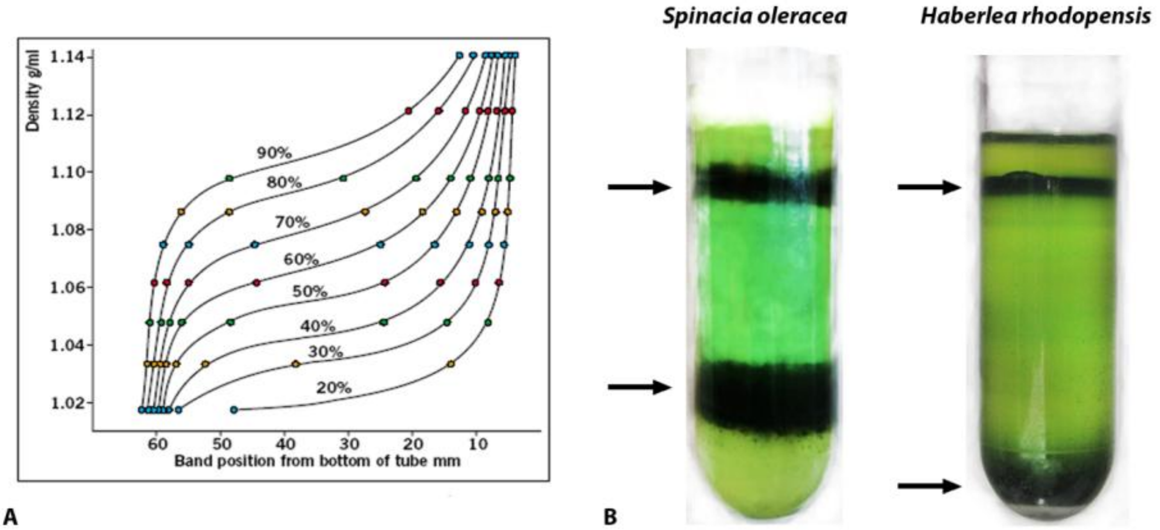

Figure S2 Comparison of chloroplasts density between *Haberlea rhodopensis* and *Spinacia oleracea.* **A)** Standard distribution of density marker beads showing gradient shape. Gradients are formed from a stock isotonic solution of Percoll shown according to their stock concentration (%). Running conditions in 23-degree angle-head rotor, 30000xg, 15 min. (Work from Amersham Biosciences, Upsala, Sweden). **B)** Distribution of broken (upper arrows) and intact chloroplasts (lower arrows) from *Spinacia oleracea* and *Haberlea rhodopensis* in 40% isotonic Percoll solution centrifuged in 23-degree angle-head rotor, 30000xg, 15 min.

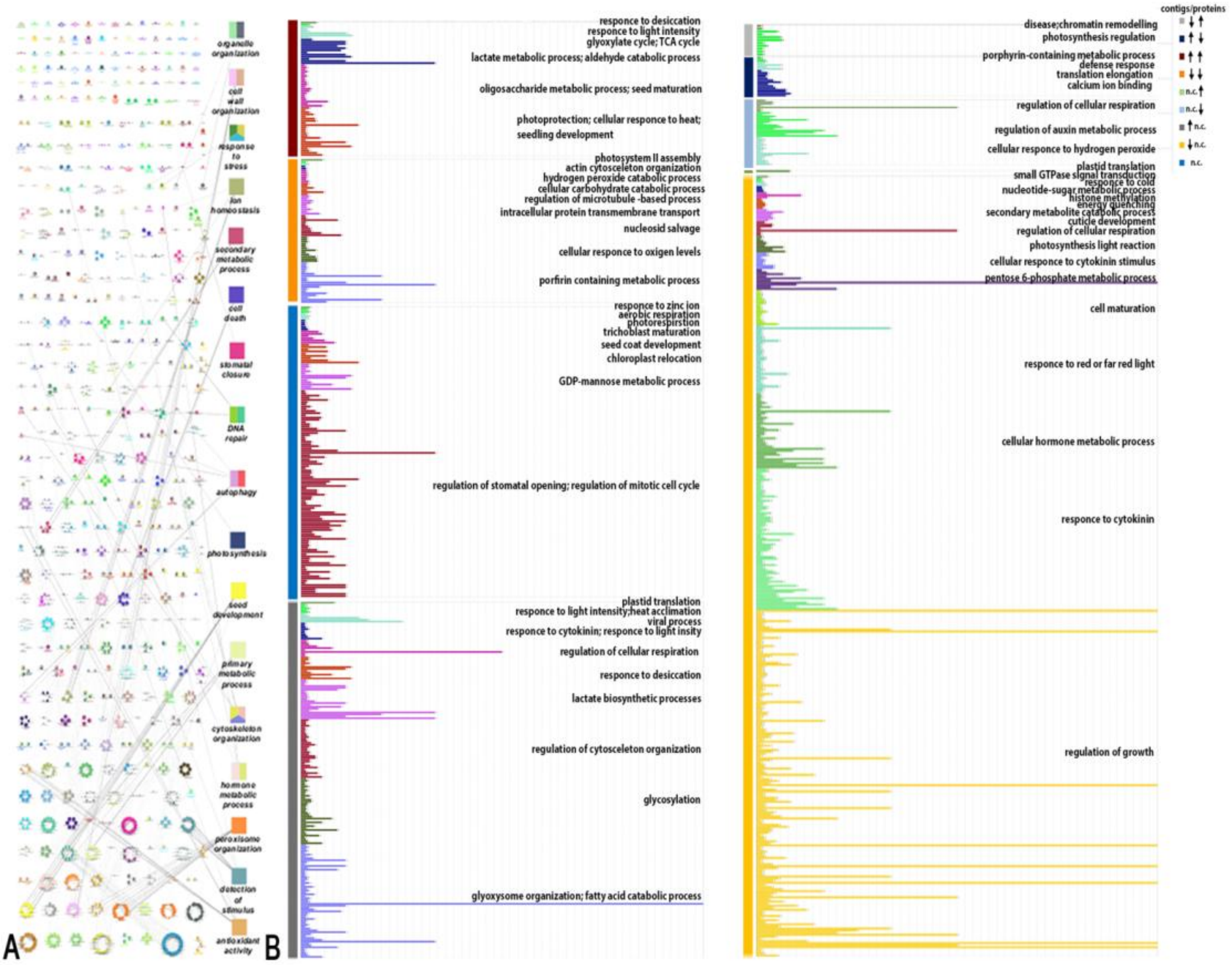

Figure S3 GO annotation of transcriptomics and proteomics data. A) CLUE GO network of all annotated RNA contigs and peptides. Processes of interest related to drought responses in the network are shown. The node color represents the type of process indicated by linkages between nodes. **B)** GO functional annotation histograms of co-expression changes of proteome and transcriptome. Bars represent the number of annotated genes to corresponding processes, functionally clustered in groups (colors). Names of significantly enriched processes for each functional group are shown. The histograms of processes are given for each co-expression pattern with a color scheme on the left corresponding to the fold changes up to 0.5 for contigs and proteins (given as color legend on upper corner).

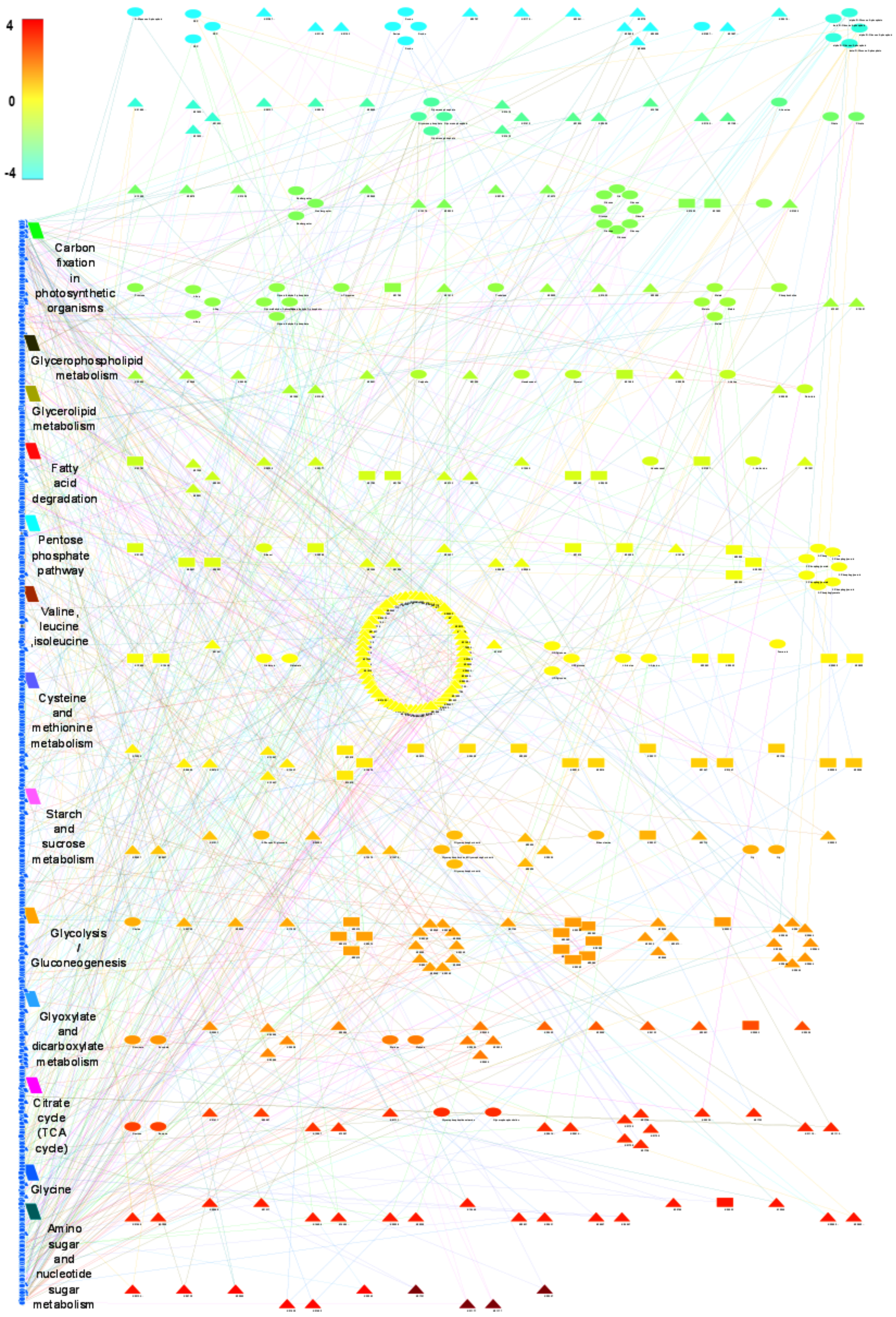

Figure S4 KEGG Network. Proteomics, transcriptomics and metabolomics data were mapped on KEGG metabolic pathways given with names and different colors on left side. Transcripts are denoted with triangles, metabolites - with ovals, and proteins - with squares. Nodes are clustered according to fold changes of values between fresh and dry states (color bar). Nodes from pathways with no assigned data were stacked in a column on the left side with blue color. Color of edges corresponding to color of nodes represents KEGG metabolic pathways.

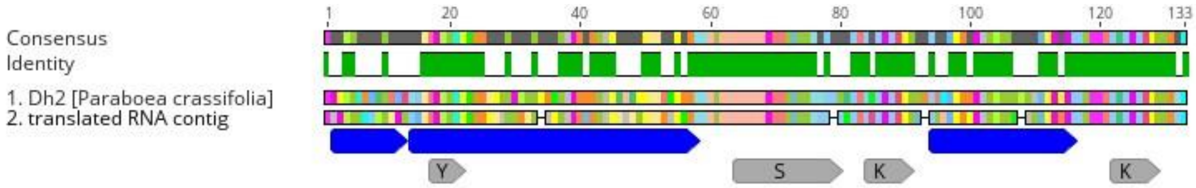

Figure S5 Alignment of identified dehydrin from *H. rhodopensis with dehydrin-like protein Dh2 [Paraboea crassifolia].* The translated RNA contig from RNA seq data from Liu et al. (2018) corresponding to identified MS peptides is aligned to the matched hit - dehydrin-like protein Dh2 [*Paraboea crassifolia*]. Consensus regions and identity are shown with annotation of the peptides identified by MS (blue). Conservative sites for dehydrins according Pfam were annotated in grey and corresponds to each dehydrin conservative segments: Y, S, K. Blast, translation, alignment and annotation are performed with Geneious Prime Software.
